# Binding of α2ML1 to the Low Density Lipoprotein Receptor-Related Protein 1 (LRP1) Reveals a New Role for LRP1 in the Human Epidermis

**DOI:** 10.1371/journal.pone.0002729

**Published:** 2008-07-23

**Authors:** Marie-Florence Galliano, Eve Toulza, Nathalie Jonca, Steven L. Gonias, Guy Serre, Marina Guerrin

**Affiliations:** 1 UMR5165 UDEAR-CNRS/UPS, CHU PURPAN, Toulouse, France; 2 Department of Pathology, University of California San Diego, La Jolla, California, United States of America; Institut Pasteur Korea, Republic of Korea

## Abstract

**Background:**

The multifunctional receptor LRP1 has been shown to bind and internalize a large number of protein ligands with biological importance such as the pan-protease inhibitor α2-macroglobulin (α2M). We recently identified *Α2ML1*, a new member of the *α2M* gene family, expressed in epidermis. α2ML1 might contribute to the regulation of desquamation through its inhibitory activity towards proteases of the chymotrypsin family, notably KLK7. The expression of LRP1 in epidermis as well as its ability to internalize α2ML1 was investigated.

**Methods and Principal Findings:**

In human epidermis, LRP1 is mainly expressed within the granular layer of the epidermis, which gathers the most differentiated keratinocytes, as shown by immunohistochemistry and immunofluorescence using two different antibodies. By using various experimental approaches, we show that the receptor binding domain of α2ML1 (RBDl) is specifically internalized into the macrophage-like cell line RAW and colocalizes with LRP1 upon internalization. Coimmunoprecipitation assays demonstrate that RBDl binds LRP1 at the cell surface. Addition of RAP, a universal inhibitor of ligand binding to LRP1, prevents RBDl binding at the cell surface as well as internalization into RAW cells. Silencing *Lrp1* expression with specific siRNA strongly reduces RBDl internalization.

**Conclusions and Significance:**

Keratinocytes of the upper differentiated layers of epidermis express LRP1 as well as α2ML1. Our study also reveals that α2ML1 is a new ligand for LRP1. Our findings are consistent with endocytosis by LRP1 of complexes formed between α2ML1 and proteases. LRP1 may thus control desquamation by regulating the biodisponibility of extracellular proteases.

## Introduction

The low density lipoprotein receptor-related protein-1 (LRP1) is a member of the low density lipoprotein (LDL) receptor family of endocytic receptors. LRP1 interacts with and internalizes a large number of protein ligands, and plays an essential role in lipid metabolism, protease/inhibitor homeostasis, and virus or toxin entry [1], [2]. Beside endocytosis, LRP1 can also regulate signaling pathways [3]. More recently, LRP1 has been directly involved in migration [4] and cancer progression [5]. LRP1 is essential for embryonic development, as blastocysts fail to transform into embryos after LRP1 targeted gene disruption in the mouse. The biological importance of LRP1 has also been highlighted by the generation of tissue-specific LRP1 knockout mice [6], [7], [8].

LRP1 is synthesized as a 600-kDa precursor protein which by proteolytic processing matures into a 515-kDa chain (α chain) and a 85-kDa chain (β chain). LRP1 has been initially described as an endocytic receptor for apolipoprotein E and for the tetrameric protease inhibitor α2-macroglobulin (α2M) [9], [10], [11]. Upon formation of a complex consisting in α2M and a protease, a conformational change within the C-terminal domain of each α2M subunit results in the exposure of a previously hidden receptor binding domain (RBD). Such an α2M molecule, designated as the activated form, is able to bind LRP1, in contrast to the native form that is not. LRP1 mediates clearance of the α2M-protease complexes by endocytosis and lysosomal degradation. As α2M is also a cytokine carrier, LRP1 may also function as a regulator of inflammation [12], [13], [14].

We recently identified a new gene of the α2-macroglobulin family, *A2ML1*, and characterized the expression of the corresponding protein, α2ML1, in the epidermis [15]. α2ML1 is expressed by keratinocytes of the uppermost granular layer of the epidermis, where it is secreted through the lamellar bodies into the extracellular space. Distinct from α2M, which is tetrameric, α2ML1 appears to be monomeric, but shares specific features of the α2M family: it presents a broad-spectrum anti-protease activity and is able to form covalent binding with proteases.

To better understand the role of α2ML1 in the epidermis, we investigated whether α2ML1 can bind LRP1. LRP1 is expressed by multiple cell types, and is especially abundant in hepatocytes, vascular smooth muscle cells, and neurons. In a study using an immunohistochemical approach, LRP1 expression was detected in skin fibroblasts and dermal dendritic cells, but was absent from the epidermis [16], while another study reported the presence of LRP1 in human epidermis and cultured keratinocytes [17].

In this study, we investigated the precise location of LRP1 in human epidermis by immunohistochemistry and immunofluorescence using two different antibodies. LRP1 appears mainly present in the granular layer of the epidermis at the periphery of the cells. We show that the putative α2ML1 RBD domain (RBDl), comprised of the 143 C-terminal residues, binds to LRP1 and is internalized with this receptor in RAW 264.7 cells. The receptor-associated protein (RAP), a protein chaperone that inhibits binding of many ligands to LRP1, inhibits binding and internalization of RBDl. Down-regulation of *Lrp1* mRNA by siRNA reduces the internalization of RBDl, demonstrating that LRP1 is required for RBDl endocytosis. Comparative amino acid and structure analysis between the RBD domains of α2M and α2ML1 together with competition experiment suggest that the binding site of α2ML1 to LRP1 may be identical from that of α2M.

## Materials and Methods

### Antibodies and Reagents

The following monoclonal (mAbs) or polyclonal antibodies were used in this study: mouse 8G1 mAb (Calbiochem), which recognizes the 515-kDa extracellular α chain of LRP1 (amino acids 1–72), mouse 5A6 mAb (Calbiochem), which recognizes the 85-kDa intracellular β chain of LRP1, polyclonal goat anti-α2M antibody (R&D Systems), polyclonal rabbit anti- pan desmocollin antibody (Serotec), polyclonal rabbit anti-involucrin antibody (BTI), anti-EEA1 mAb (BD Transduction Laboratories), anti-GST mAb (Pierce), anti-actin mAb and MOPC IgG2 mAb (Sigma). The polyclonal rabbit anti-corneodesmosin was described elsewhere [18]. SiRNA duplexes were purchased from Qiagen (MmLrp1-1 siRNA, MmLrp1-7 siRNA and Allstars negative Control siRNA). Streptavidin peroxidase and streptavidin fluorescein were from Boehringer Mannheim. TRITC conjugate goat anti-mouse antibody was from Immuonotech. Alexa 488 conjugate goat anti-mouse and 555 goat anti-rabbit antibodies were from Invitrogen. GST-RAP was described elsewhere [19]. Activated human α2M (α2M-MA) was from BioMac.

### Biological materials

All human skin samples were obtained from donors undergoing plastic surgery (Dr JP Chavoin) after informed verbal consent, as recommended by the local ethics committee (CHU Toulouse, France), and in accordance with Helsinki principles.

### Production of recombinant RBDl

A cDNA fragment encoding the last 143 amino-acids of α2ML1 (aa 1312–1454 GenBank NP_653271, denoted RBDl) was PCR-amplified and subcloned into PGEX6p1 (Amersham Biosciences). The construct was transformed into BL21-codonPlus bacteria (Stratagene). The extraction of the recombinant GST-RBDl fusion protein was essentially performed according to the online protocol contributed by Dr. Chia Jin Ngee. Basically, after lysozyme digestion, proteins from cell lysates were solubilized in 0.7% Sarkosyl and 2% triton X-100 in 10 mM Tris-HCl, pH 8, 1 mM EDTA, and 150 mM NaCl. RBDl was purified by affinity on a glutathione sepharose column and eluted by 10 mM glutathione, pH 8. The recombinant protein was dialyzed against PBS and quantified using a standard BioRad assay. Using a similar procedure, a cDNA fragment encoding 148 amino acids positioned in the central bait region of α2ML1 (aa 586–734, denoted CBD) was used to produce a GST fusion protein. All plasmid constructs were checked by sequencing performed by standard procedures.

### Cell culture

The murine macrophage-like RAW 264.7 cell line was grown in DME/Glutamax medium supplemented with 10% SVF and antibiotics.

### Immunoblotting

RAW cells were lysed in RIPA buffer (50 mM Tris-HCl, pH 7.5, 150 mM NaCl, 10 mM EDTA containing 0.1% SDS, 1% Triton X-100, 0.5% Na deoxycholate and a protease inhibitor cocktail (Sigma)). Epidermal proteins were extracted with 40 mM Tris-HCl, pH 7.5 and 10 mM EDTA containing 0.5% Nonidet P-40 and protease inhibitors. Biochemical analysis of LRP1 expression was performed by immunoprecipitation assays using either 8G1 mAb (1 µg/ml) or 5A6 mAb (1 µg/ml). Incubation was performed overnight at 4°C under agitation. Protein A/G sepharose beads (Pierce) were then added and an additional incubation of 1 h was performed at 4°C. After three washes in RIPA buffer, Laemmli buffer without reducing agents was added to the sepharose beads. Samples were analyzed by western blotting using standard procedures.

### Binding experiments

RAW cells grown to 80% confluence in T25 flasks were washed abundantly with OPTI-MEM medium and incubated with 5 µg/ml of RBDl or control protein CBD for 2 h at 4°C under gentle agitation. After extensive washes, cells were lysed on ice by adding 1 ml of RIPA buffer. Genomic DNA was disrupted by several passages through a needle. Immunoprecipitation was carried out by adding 1 µg of 8G1 or MOPC mAbs using the procedure described above. The membrane was probed with anti-GST mAb (1:10 000). In some experiments with RAP, the cells were preincubated or not for 30 min at 4°C with GST-RAP at 5 µM before addition of RBDl or CBD for 2 h at 4°C. Immunoprecipitation was carried out by adding 1 µg of anti-GST mAb followed by western blot with anti-GST. For competition experiment, RAW cells were preincubated for 30 min at 4°C with a range of RBDl (0.05, 0.1, 0.5 and 1 µM) or with GST (1 µM) before α2M-MA was added onto the cells at a concentration of 0.015 µM and incubated for 2 h at 4°C. Bound α2M-MA was immunoprecipitated with anti-α2M (0.5 ug/ml) and detected with the same antibody by western blot.

### Uptake experiments

RAW cells were plated 24 h before the experiments in 6-well plates at a density of 400,000. Cells were washed with pre-warmed OPTI-MEM medium and incubated with RBDl, GST alone, CBD protein (each at 35 nM) or activated α2M-MA (14 nM) at 37°C for different times. Cells were then washed abundantly and lysed in 1 ml of RIPA buffer. Immunoprecipitations were performed as described before with anti-GST mAb (1 µg/ml) or anti-α2M antibody (0.5 µg/ml). The immunoprecipitated proteins were analyzed by Western blot with anti-GST or anti-α2M antibodies.

### LRP1 gene silencing

siRNA transfection was performed according to the Qiagen supplementary protocol for macrophage cell lines. Two mouse specific *Lrp1* siRNAs (Lrp1.1 and Lrp1.7) or negative control siRNA (AllStars Negative Control) were transfected. The targeted sequences of *Lrp1* (NM_008512) are: _(nt5945)_ CACGTTGGTTATGCACATGAA_(nt5965)_ for Lrp1.1 siRNA and _(nt5498)_ CACCAACAAGAAGCAGATTAA_(nt5518)_ for Lrp1.7 siRNA. The negative control siRNA had no target sequence. The day before transfection, 100,000 cells were seeded in 24-wells and then transfected in triplicate using 50 nM siRNA with 6 µl of reagent buffer (HiPerfect, Qiagen). Gene silencing was analyzed by quantitative real time PCR 48 h after transfection. For RBDl uptake quantification, triplicates were lysed in RIPA buffer and pooled before immunoprecipitation with anti-GST. After precipitation of the sepharose beads, the supernatants were used as loading controls to monitor total protein content by western blot with an anti-actin mAb.

### Real-time qPCR

Total RNA from triplicate wells was extracted using the RNeasy extraction kit (Qiagen). Reverse transcription was performed via a standard procedure, using 1.5 µg of total RNA and a mixture of oligo(dT) and random primers. Two sets of primers were chosen using Primer3 software [20] for the amplification of *Lrp1* exons 41–42 (amplifying a 137 bp fragment) and exons 76–77 (amplifying a 105 bp fragment). Sequences were as follows: exons 41–42 forward 5′-ACTTTGGGAACATCCAGCAG-3′ and reverse 5′-GGTGGATGTGGTGTAGCTTG-3′; exons 76–77 forward 5′-CCCTCCTACCACTTCCAACC-3′ and reverse 5′-CCCAGTCGATAGCGATACC-3′. A pair of primers specific for the housekeeping gene mouse beta-2 microglobulin (*B2m*) generating an amplicon of 166 bp (forward 5′-ACGCCTGCAGAGTTAAGCAT-3′ and reverse 5′-GCTATTTCTTTCTGCGTGCAT-3′) was used as the normalizer for each sample. Amplifications were performed with the ABI prism 7300 Sequence Detection System and analyzed with the corresponding software (Applied Biosystems) using the qPCR ROX-&GO Green mix (MP Biomedicals). All amplified products were checked by dissociation curve analysis. Samples were analyzed in triplicate and quantified using the comparative Ct method [21]. Threshold cycle (Ct) values for *Lrp1* were normalized to Ct values for *B2m*. The relative *Lrp1* mRNA level (expressed in %) was calculated from the mRNA ratio of either Lrp1 siRNA-transfected cells vs untransfected cells or negative control siRNA-transfected cells vs untransfected cells. Standard deviations were calculated using the methods of standard propagation of error.

### Immunohistochemistry and immunofluorescence

Immunohistochemistry was performed on formaline-fixed skin samples embedded in paraffin using regenerator buffer S2368, pH 9.0 and either the K5001 kit when using 8G1 mAb or the Strept.ABC kit when using 5A6 mAb (both from DAKO). Samples were incubated at room temperature with 8G1 mAb (2 µg/ml) and with 5A6 mAb (10 µg/ml). Negative controls were incubated with secondary antibody alone. For immunofluorescence, cryosections of human skin samples were blocked in PBS, BSA 1%, Tween-20 0.2% for 1 h. All incubations and washes were performed at room temperature in the same blocking buffer. Incubations were as follows: primary antibody (8G1 or 5A6 mAbs, 2 µg/ml) for 2 h, Alexa 488 conjugate goat anti-mouse antibody (green) for 1h. For double staining, the same procedure was followed by incubations with polyclonal rabbit antibodies and then with Alexa 555 conjugate goat anti-rabbit antibody (red). Imaging was performed using a Leica fluorescence microscope and NIS-Elements BR2.30 software.

### Internalization of biotinylated RBDl and confocal microscopy

Recombinant RBDl protein or control GST (150 µg each) were incubated with NHS-Biotin (PIERCE) with a molar ratio biotin: recombinant protein of 40:1 in 0.1 M NaHCO_3_, pH 8 for 1 h at room temperature. The reactions were stopped by addition of L-Lysine to a final concentration of 0.5 mM followed by an additional incubation for 30 min. The proteins were then dialyzed against PBS pH 7.4. Biotinylation was controlled by loading 1 µg of the proteins on gels followed by western blotting with streptavidin-peroxidase.

RAW cells were plated on glass cover slips at a density of 50,000/cm^2^. After overnight recovery, the cells were incubated in the presence of 60 nM biotinylated protein in OPTI-MEM medium for 30 min at 37°C or for 1h at 4°C. The cells were then washed and fixed with methanol for 5 min at −20°C. Blocking buffer containing streptavidin-fluorescein (2 µg/ml) was then added for 1 h. For double labeling, incubation with 8G1 (2.5 µg/ml) or anti-EEA1 (1∶200) mAbs followed by TRITC goat conjugate anti-mouse antibody took place before labeling with streptavidin-fluorescein. Analyses were performed by confocal imaging. In experiments using RAP, the cells were preincubated with 5 µM GST-RAP for 30 min at 37°C before addition of biotinylated RBDl, followed by incubation for 30 min at 37°C. In experiments with siRNA-transfected cells, biotinylated RBDl was added to untransfected cells, mock siRNA or LRP1siRNAs-transfected cells 48 h after transfection. The procedure described above was then followed. Imaging was performed using a Leica fluorescence microscope and NIS-Elements BR2.30 software.

### Structure predictions and alignments

Secondary structure of RBDl was predicted using the New Joint method-based PAPIA package (http://mbs.cbrc.jp/papia/papia.html) and by comparison with the NMR structure of RBD of α2M [22], [23]. A 3D model of RBDl was built using as a template the 3D structure 1BV8, which corresponds to human RBD, and by using the DeepView-The Swiss-PdbViewer software (http://www.expasy.org/spdbv/). RBD alignments were performed using the Multalin algorithm (http://www-archbac.u-psud.fr/genomics/multalin.html). Orthologs of α2ML1 were as follows: chimpanzee (XP_520828), rhesus monkey (XR_014195), dog (XP_543824), cow (translated from EST CB226612). Predicted cat and hedgehog α2ML1 orthologs were translated from genomic sequences.

## Results

### LRP1 is expressed in the granular layer of the human epidermis

**Figure 1:**
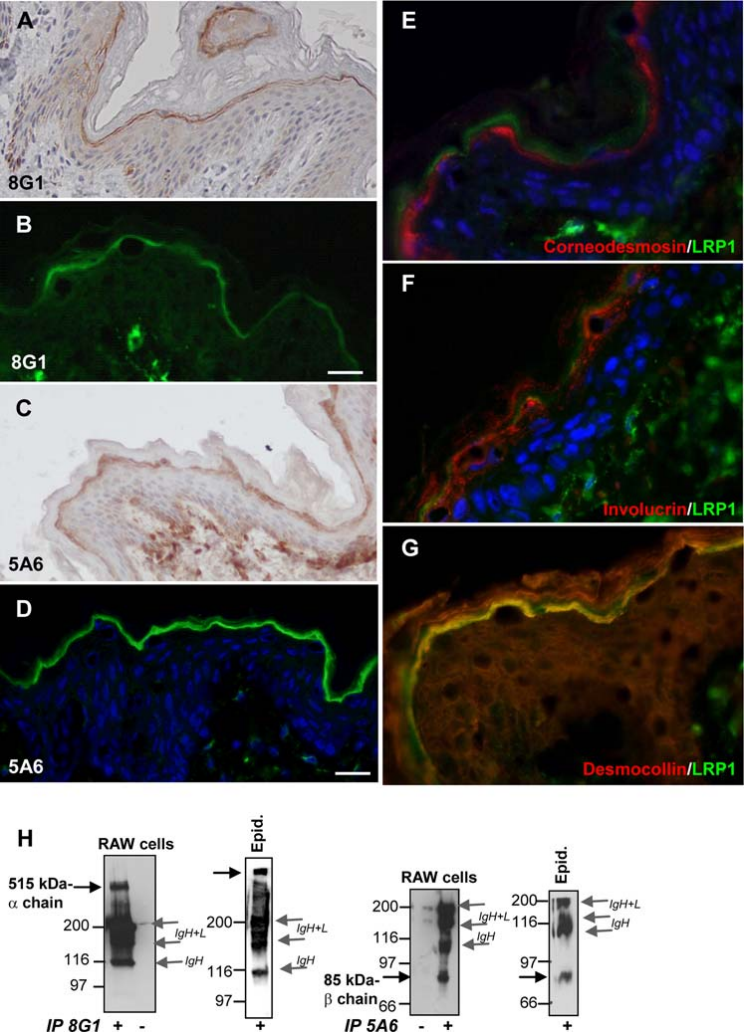
Expression of LRP1 in human epidermis. Immunohistochemistry and immunofluorescence analyses on skin samples in the presence of 8G1 (A, B) or 5A6 mAbs (C–G). A, the α chain of LRP1 labeling shows weak cytoplasmic staining in the spinous layers while it appears to locate at the periphery in the upper layers of the epidermis. B, using immunofluorescence, the α chain of LRP1 labeling is detected in the granular layer of epidermis. The dermis was also labeled. C and D, the β chain of LRP1 is associated within the granular layer of epidermis. The dermis was positive. A, C, original magnification×200. B, D, bar, 15 µm. E–G, Double labeling for LRP1 and corneodesmosin (E), involucrin (F) and desmocollin (G). LRP1 does not colocalize with corneodesmosin or involucrin but colocalizes with desmocollin 1 within keratinocytes of the granular layer. D, E, F, nuclei were counterstained with TOTO. H, Biochemical analysis of LRP1 expression. The α chain and β chain of LRP1 were detected in RAW cells and in human epidermis by immunoprecipitation. Lysates from RAW cells or 300 µg of epidermal proteins (epid.) were incubated with 8G1 or 5A6 mAbs or without antibody (−). Standard immunoprecipitations were then applied. The blots were probed with the same antibodies. Under non-reducing conditions, the dimers formed by IgH and IgL chains were detected.

To determine LRP1 expression in the course of keratinocyte differentiation, we performed immunohistochemistry and immunofluorescence analyses (Figure 1A–G). We used either 8G1 or 5A6 mAbs, which recognize the α chain and the β chain of LRP1, respectively. Immunohistochemistry (A, C) and immunofluorescence (B, D) revealed that LRP1 is mainly expressed in the granular layer of the epidermis. The granular layer is the uppermost layer of living cells beneath the stratum corneum and is constituted by the most differentiated keratinocytes. We also detected LRP1 in dermal fibroblasts with the same mAbs, a finding in agreement with previous studies [17]. We then performed double staining with three well-known markers for differentiation of epidermal keratinocytes, namely corneodesmosin (late marker, cytoplasmic and secreted, specific of the granular layer) (Figure 1E), involucrin (early marker, cytoplasmic) (Figure 1F), and with the desmosome protein desmocollin 1 (late marker, transmembrane, specific of the granular layer) (Figure 1G). The labeling for involucrin and corneodesmosin did not superimpose with that of LRP1 within keratinocytes of the granular layer. Detection of the epidermal isoforms of the transmembrane desmosome protein desmocollin [24] with a pan anti-desmocollin antibody allowed detection of the desmocollin 2 and 3 isoforms in basal keratinocytes, and of the desmocollin 1 isoform in the keratinocytes of the granular layer (Figure 1G). As anticipated, the desmocollin staining was pericellular, with a strong and polarized staining for desmocollin 1 within the granular layer towards the upper face of the stratum corneum. Co-localisation of LRP1 with desmocollin 1 within the granular layer suggested that LRP1 is located at the periphery of keratinocytes.

Analysis of LRP1 by Western blot was carried out on protein extracts from either epidermis or the macrophage-derived RAW 264.7 cell line, which is known to express LRP1 as a functional receptor [25]. In RAW cells, but also in human epidermis both the α chain and the intracellular β chain were detected after immunoprecipitation with 8G1 and 5A6 mAbs, respectively (Figure 1H). Altogether, these findings suggest that LRP1 is a functional receptor in the upper layers of the epidermis.

### The RBD domain of α2ML1 is internalized into RAW cells and binds to LRP1 at the cell surface

**Figure 2:**
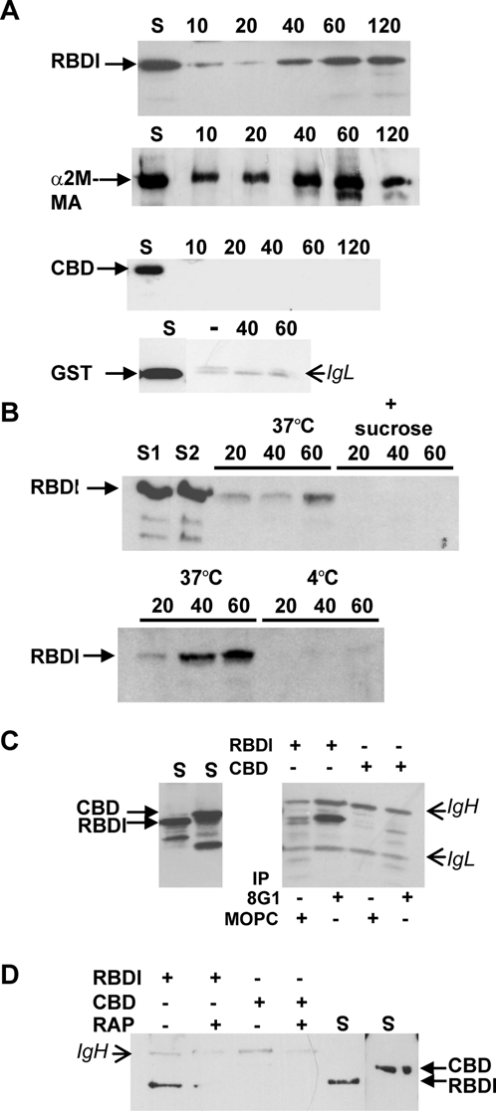
Analysis of the interaction of RBDl with LRP1. A, Uptake of RBDl by RAW cells. RAW cells were incubated or not (−) with RBDl, α2M-MA as a positive control or GST as a negative control for the indicated periods of time. Immunoprecipitations were carried out with anti-GST or anti-α2M antibodies. Samples were analyzed by immunoblot with the same antibodies. RBDl was internalized in a time dependant manner. As anticipated, the activated form of α2M was internalized while GST was not. S, supernatant recovered from cell culture medium after incubation. IgL indicates the light chain of immunoglobulins. B, endocytosis-dependent uptake of RBDl. RAW cells were incubated with RBDl in the presence or absence of 0.4 M sucrose at 37°C, or at 4°C versus 37°C in parallel experiments for the indicated periods of time. Immunoprecipitations were performed as described above. Addition of sucrose or incubation at 4°C blocked the uptake of RBDl. S1 and S2 are supernatants recovered from cell culture medium after incubation of RBDl in the absence or presence of sucrose, respectively. C, Binding of RBDl to LRP1. RAW cells were incubated in the presence of RBDl or CBD for 2 h at 4°C. Aliquots of the supernatants (S) were then collected and the cells were lysed. Immunoprecipitation was performed using the anti LRP1 8G1 mAb or the MOPC antibody as control. Co-immunoprecipitated proteins were detected by Western blot using the anti-GST antibody. RBDl was specifically co-immunoprecipitated by anti-LRP1 antibody. IgH and IgL indicate the heavy and the light chains of immunoglobulins. D, RBDl binding at the cell surface in the presence of RAP. RAW cells were preincubated or not with GST-RAP for 30 min at 4°C before addition of RBDl or CBD for an additional 2 h incubation at 4°C. Immunoprecipitation and Western blot detection was performed using the anti-GST mAb. RBDl, but not CBD, was found associated at the cell surface in the absence of GST-RAP. Addition of GST-RAP inhibited RBDl binding to the cells. S, supernatants loaded as control.

It has been shown that the RBD domain of α2M is solely responsible for the binding of α2M-proteinase complexes to LRP1 [26] [23]. Many studies of α2M binding to LRP1 have used recombinant proteins that represent the carboxy-ends of α2Ms expressed in bacteria [19], [27], [28]. By homology with the RBD domain of α2M, we defined the potential RBD domain of α2ML1. This domain, denoted RBDl, was produced as a GST-fusion protein. We first asked whether the RBDl protein could be internalized into RAW cells, as those cells have been described by others as expressing LRP1 at high levels [25]. Uptake experiments were performed by incubating RBDl, GST alone as a negative control, or the activated form of α2M (α2M-MA) as a positive control, onto RAW cells at 37°C for different times. Immunoprecipitations of recombinant proteins from the cell extracts were then analyzed. RBDl was internalized in a time-dependent fashion into RAW cells, and this was similar to the uptake of the activated form of α2M albeit with a slower kinetic (Figure 2A). The GST protein alone as well as the central bait domain of α2ML1 (recombinant protein CBD) were not internalized. To confirm that RBDl uptake was specific and mediated by endocytosis, we carried out, in parallel, an incubation of RBDl in the presence of 0.4 M sucrose, which has been shown to inhibit clathrin-dependent endocytosis [29], [30] (Figure 2B). Additionally, to exclude the hypothesis that immunoprecipitated RBDl only resulted from binding at the cell surface during incubation, parallel incubations of RBDl were performed at 4°C. As shown in Figure 2B, sucrose prevented the uptake of RBDl, supporting the hypothesis that RBDl is internalized by endocytosis. When incubation was performed at 4°C, RBDl was poorly detectable, thus revealing that in our experimental conditions for RBDl uptake, immunoprecipitated RBDl mainly corresponds to the protein that has been internalized. It should be noted that the relative high level of RBDl detected at 37°C as compared to the level of cell-associated RBDl at 4°C may account for accumulation of the protein into the cells before degradation. We then asked whether RBDl could be coimmunoprecipitated with LRP1. Binding experiments were performed by incubating RBDl onto RAW cells in serum-free medium for 2 h at 4°C to prevent internalization, followed by immunoprecipitation of LRP1 by 8G1 mAb. As shown in Figure 2C, RBDl was coimmunoprecipitated by 8G1 mAb, but not by the irrelevant MOPC mAb. Moreover, the CBD protein did not coimmunoprecipitate with the LRP1 antibody. Thus, the specific immunoprecipitation of RBDl together with LRP1 suggests that RBDl interacts with LRP1. To further demonstrate the specificity of interaction between RBDl and LRP1, we analyzed the binding of RBDl at the cell surface in the presence of RAP, a universal ligand competitor for LRP1 [19], [31]. In this experiment (Figure 2D), GST-RAP at 5 µM was added onto the cells for 30 min at 4°C before addition of RBDl or CBD. After 2 hours of incubation at 4°C, RBDl binding at the cell surface was clearly detected. When GST-RAP was present, RBDl was no longer immunoprecipitated, supporting that LRP1 was involved in RBDl binding at the cell surface.

### The RBD domain of α2ML1 colocalizes with LRP1 upon internalization *in vivo*

**Figure 3:**
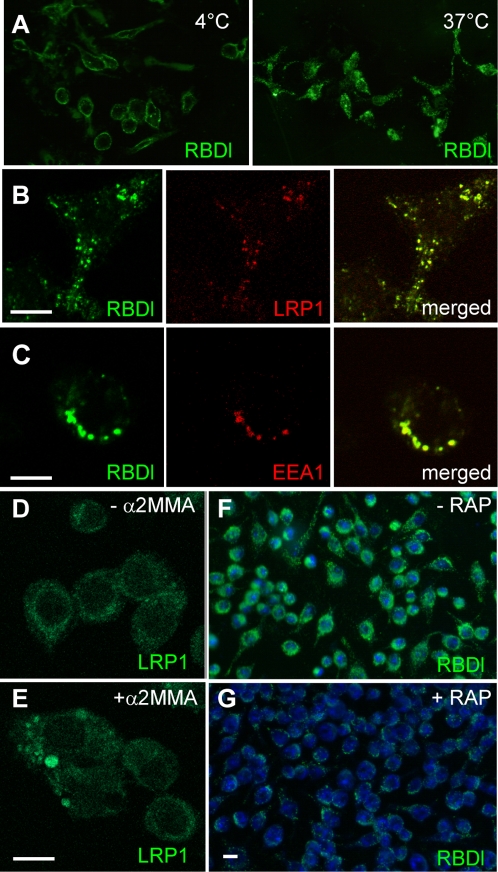
Analysis of internalization of biotinylated RBDl into RAW cells. A, Representative pictures obtained with streptavidin-fluorescein labeling. A membrane staining was pronounced when cells were incubated with RBDl for 1 h at 4°C. A cytoplasmic staining with punctuate appearance was observed when cells were incubated with RBDl for 30 min at 37°C. B,C, confocal pictures of individual cells depicting double staining with streptavidin-fluorescein and with either 8G1 mAb (B) or anti-EEA1 mAb (C) coupled withTRITC conjugate anti-mouse antibody. RBDl co-localized with LRP1 and with EEA1 in the cytoplasm. D, E, Immunofluorescence labeling of LRP1 with 8G1 mAb followed by Alexa-488 conjugate anti-mouse antibody on RAW cells either untreated (D) or incubated with α2M-MA (E) for 37°C at 30 min. A cytoplasmic staining with clustering appearance was observed when cells were incubated with α2M-MA. F, G, streptavidin-fluorescein labeling with TOTO nuclear counterstain of cells preincubated or not with GST-RAP for 30 min at 37°C before addition of biotinylated RBDl for 30 min at 37°C. The biotinylated RBDl was poorly internalized into the cells in the presence of GST-RAP. Pictures were taken with the same time exposure (137 ms). Bars, 5 µm.

To confirm and investigate internalization of RBDl via LRP1, we analyzed the binding and internalization of a biotinylated form of RBDl onto RAW cells by confocal microscopy using strepatavidin-fluorescein conjugate for detection. We first confirmed the binding of RBDl to the cell membranes by incubation of the biotinylated RBDl at 4°C to prevent internalization. As expected, cell membranes were labeled by the biotinylated RBDl protein (Figure 3A, left panel). Incubation at 37°C for 30 min induced a shift of the biotinylated RBDl from the cell membrane to the cytoplasm of the cells (Figure 3A, right panel). The cluster appearance of the labeling was suggestive of formation of endosomal vesicles. A biotinylated control GST, when incubated at 37°C, was not detectable in the cytoplasm of the cells (not shown). Double staining with 8G1 mAb revealed that LRP1 colocalized with RBDl in intracellular vesicles (Figure 3B). When double staining was performed with an anti-EEA1 mAb, RBDl was also found to colocalize with EEA1, a marker of early endosomes (Figure 3C). We noted that a similar clustering appearance of LRP1 was observed when α2M-MA was incubated on cells for 30 min at 37°C (Figure 3D, E). In another set of experiments, internalization of biotinylated RBDl was compared between cells that had been preincubated with 5 µM GST-RAP for 30 min at 37°C and those that had not before incubation with biotinylated RBDl for 30 min at 37°C (figure 3F, G). While RBDl was strongly detected in the cytoplasm of the control cells, poor labeling was observed within cells that had been preincubated with GST-RAP. In conclusion, and consistent with the *in vitro* experiments, our results indicate that RBDl is specifically internalized into RAW cells by endocytosis and that LRP1 is involved in this process.

### Silencing Lrp1 with siRNA reduces the internalization of the RBD domain of α2ML1

**Figure 4:**
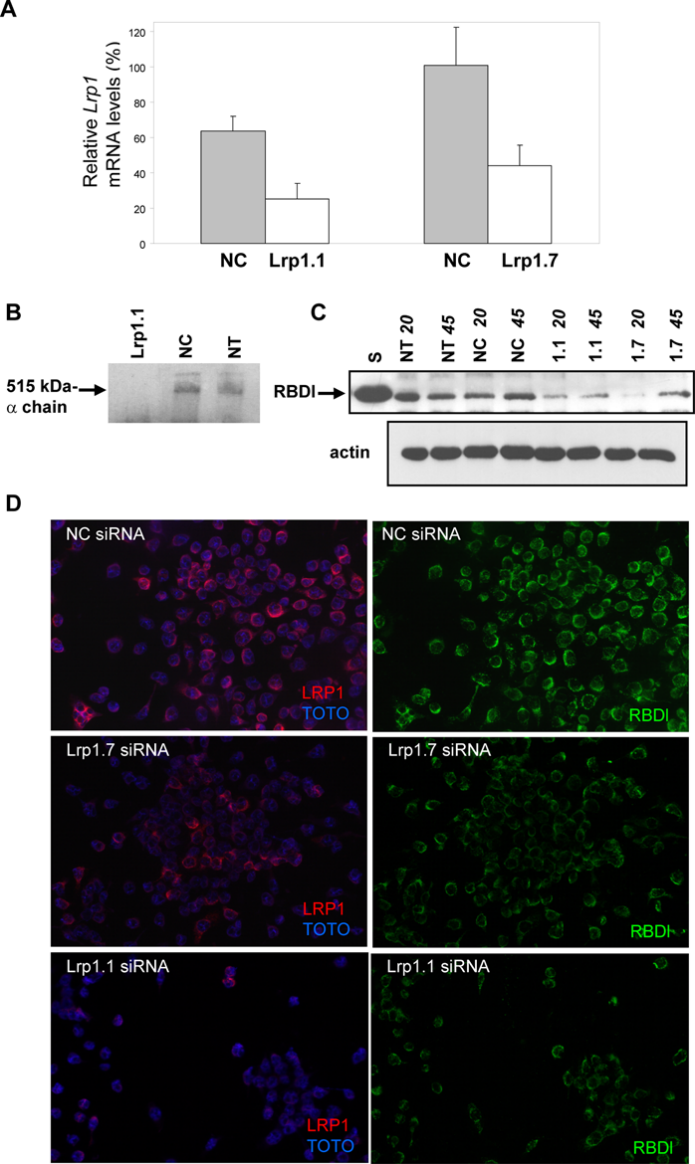
Analysis of RBDl internalization in Lrp1-deficient cells. RAW cells were transfected or not (NT) with one of the two Lrp1-siRNAs, Lrp1.1 or Lrp1.7, or with an unrelated siRNA as negative control (NC). 48h after transfection, *Lrp1* mRNA was determined by real time qPCR (A), at the protein level (B) or the cells were challenged for RBDl uptake (C, D). A, *Lrp1* mRNA is reduced in Lrp1-siRNAs transfected cells. B, LRP1 is down-regulated at the protein level in Lrp1-siRNA transfected cells. Immunoprecipitation of the α chain was performed using 8G1 mAb. The α chain was detectable in NT and NC cells but not in Lrp1.1-siRNA cells. C, RBDl uptake by LRP1-deficient cells is reduced. The cells were incubated with RBDl for 20 or 45 min. Uptake of the protein was analyzed by immunoprecipitation followed by immunoblot with the anti-GST mAb. S, supernatant recovered from cell culture medium after incubation. Total protein contents were monitored by Western blotting with a β-actin antibody. The blot shown was representative of two independent transfections. D, Internalization of biotinylated RBDl into Lrp1-deficient cells is compromised. NC siRNA-transfected cells, Lrp1.1 and Lrp1.7 siRNA-transfected cells were incubated with biotinylated RBDl for 30 min at 37°C. Pictures depict streptavidin-fluorescein labeling (green) or anti-LRP1 5A6 mAb labeling (red) with TOTO nuclear counterstain. Bar, 10 µm. Time exposures were 130 ms for overall pictures.

To investigate whether LRP1 is required for the uptake of RBDl, we used a siRNA approach to downregulate *Lrp1* expression in RAW cells. Cells were transfected with two mouse Lrp1-specific siRNAs (Lrp1.1 and Lrp1.7), a negative control siRNA (NC), or were untransfected (NT). *Lrp1* abundancy was assessed at the mRNA level by real time quantitative RT-PCR using the relative quantity method and NT cells as the calibrator. Both Lrp1-siRNAs significantly decreased *Lrp1* mRNA level (Figure 4A), while no significant change was observed in NC cells. Western blot detection of LRP1 after immunoprecipitation with the 8G1 mAb shows that the α chain of LRP1 was not detectable in cells transfected with Lrp1.1-siRNA (Figure 4B). RBDl uptake in siRNAs-transfected cells was analyzed both by biochemical experiments (Figure 4C) and immunofluorescence analysis (Figure 4D). As shown in Figure 4C, the internalization of RBDl was significantly reduced in cells transfected with Lrp1-siRNAs by comparison with the uptake of RBDl into non-transfected cells (NT) or into cells transfected with NC siRNA. In the same manner, the uptake of biotinylated RBDl was clearly reduced in Lrp1 siRNAs-transfected cells as compared to NC-transfected cells, and this was correlated with the reduced expression of Lrp1 revealed by 5A6 mAb labeling (Figure 4D). Altogether, these data demonstrate that LRP1 is required for RBDl internalization by RAW cells.

### Comparative structure analysis and competition assay between α2ML1 and α2M suggest a similar mechanism for binding to LRP1

**Figure 5:**
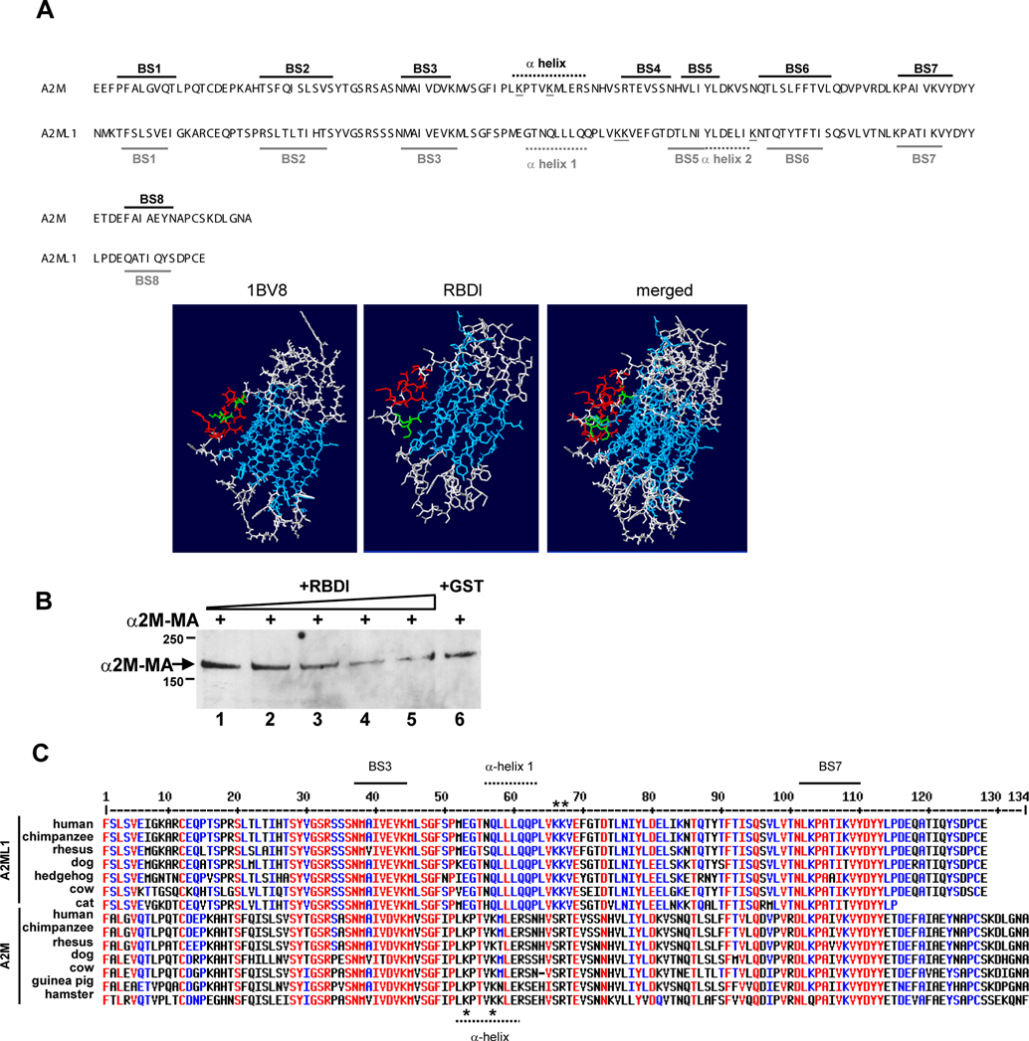
Predicted structure of RBDl. A, Comparison of the NMR-determined secondary structure for human α2M RBD (according to reference 23) with the predicted secondary structure for human α2ML1 RBDl. The heavy lines with the letter “BS” indicate regions of β sheet conformation and the dotted lines indicate regions of α-helical conformation (black color for α2M and grey color for α2ML1). The α-helix region of α2M RBD is assumed to trigger binding to LRP1. The two lysine residues (Lys1370 and Lys1374) are underlined. In α2ML1 RBDl, two distinct α-helices (denoted 1 and 2) are predicted, and the three lysine residues surrounding those regions are underlined. The S4 β-sheet is missing while the S5 β-sheet is only predicted by the method of Chou-Fasman. A hypothetical 3D model of RBDl was built using the 3D structure 1BV8 (human RBD) as template. The major helix regions are labeled in red and the β-strands are labeled in blue. Lysine residues (Lys1370 and Lys1374 for 1BV8, and Lys1392–1393 for RBDl) are labeled in green. B, α2M-MA binding at the cell surface in the presence of RBDl. RAW cells were preincubated or not (lane 1) with RBDl (lanes 2 (0.05 µM), 3 (0.1 µM), 4 (0.5 µM), 5 (0.5 µM)) or with 1 µM of GST protein (lane 6) for 30 min at 4°C before addition of α2M-MA at 0.015 µM for an additional 2 h incubation at 4°C. Immunoprecipitation and Western blot detection was performed using anti-α2M antibody. RBDl competes for α2M-MA binding in a dose-dependent manner. C, Multalin RBD alignments between representative α2M members, human α2ML1 and predicted orthologs of α2ML1. The dotted lines indicate the α-helix domains and lysine residues are marked by asterisks.

RBD of α2M contains two lysine residues that are important for binding to LRP1 [27], [28], [32], [33]. These two lysine residues are highly conserved among macroglobulins of the α2M family [28], suggesting a common mechanism for binding to LRP1. Remarkably, α2ML1 lacks the two corresponding lysine residues. Therefore we attempted to define a predicted structure of RBDl As represented in Figure 5A, the predicted secondary structure of RBDl shows overall similarity with the RBD structure. Distinctively, the major α-helix segment containing the two key lysine residues in α2M is missing in α2ML1, while two distinct α-helix domains (denoted α-helix 1 and α-helix 2) are located between the BS3 and BS6 β-sheets. A hypothetical 3D model of RBDl was built using as template the 3D structure 1BV8, which corresponds to human RBD. As represented in Figure 5A, the RBDl 3D model closely resembles the 3D structure of human RBD, suggesting that both proteins may bind to the same binding site of LRP1. To adress this question, we analyzed the binding of α2M-MA at the surface of RAW cells in the presence of increasing amounts of RBDl. In this experiment, RBDl (0.05, 0.1, 0.5 and 1 µM) was added onto the cells for 30 min at 4°C before addition of α2M-MA at 0.015 µM followed by an additional incubation for 2 hours at 4°C. RBDl reduced the binding of α2M-MA in a dose-dependent manner (Figure 5B), suggesting that RBDl competed for α2M-MA binding to LRP1. GST protein at 1 µM had no significant effect on α2M-MA binding.

The Alignment of RBD sequences of A2M proteins and predicted A2ML1 orthologs allowed us to delineate regions conserved among both groups from group-specific regions (Figure 5C). For example, the regions that constitute the BS3 and BS7 β-sheets are highly conserved in all sequences. In contrast, the α helical domains are not conserved between the two groups. Notably, the predicted α-helix 1 is conserved in all A2ML1 orthologs. Interestingly, the two lysine residues positioned in tandem in close vicinity with the α-helix 1 are conserved, suggesting that these residues may be important for the binding to LRP1.

## Discussion

In this study, we demonstrate that the Receptor Binding Domain of α2ML1 (RBDl) interacts with and is internalized by LRP1. By down-regulating *Lrp1* expression, we show that LRP1 is necessary for RBDl internalization. Our finding suggests that α2ML1, like many other macroglobulins, is a ligand of LRP1. Distinctively, α2ML1 misses the exposed α helix region of the RBD domain of α2M that contains the two lysine residues implicated in binding to LRP1. However, the predicted 3D model of RBDl indicates that α2ML1 may bind to the same site of LRP1 than α2M does [33]. Indeed, RBDl contains a distinct, and conserved, predicted α-helical region of which two lysine residues are positioned in close vicinity. In the future, it will be interesting to further delineate the region of RBDl required for binding to LRP1.

Thus far, the expression of LRP1 in the epidermis has been controversial [16], [17]. Here, we validate the presence of LRP1 in human epidermis. Using immunohistochemistry and immunofluorescence, we found that LRP1 is expressed in the granular layer of epidermis where it appeared to locate at the plasma membrane. Previously, Birkenmeir *et al* had used immunofluorescence labeling and found expression of LRP1 throughout the epidermis with pronounced labeling in the basal layer of epidermis [16], [17]. The discrepancy with our immunohistochemistry and immunofluorescence analyses may be explained by the use of different antibodies. However, the mAb used by Birkenmeir is no longer commercially available. We believe that the concordant staining we obtained with both the 8G1 and 5A6 mAbs support our conclusions. In agreement with Birkenmeir, we found by immunofluorescence analysis that LRP1 was expressed in the cytoplasm of actively proliferating primary keratinocytes grown *in vitro.* Also, we observed that LRP1 labeling was more intense at the periphery of the cells when keratinocytes were induced to differentiate by 48 h exposure to 1.5 mM calcium (our observations). It will be interesting to study the regulation of LRP1 expression during the differentiation of keratinocytes.

We have previously shown that α2ML1 is expressed specifically in the granular layer of keratinocytes and is secreted into the extracellular space between the granular layer of keratinocytes and the uppermost layer of corneocytes [15]. *In vitro*, α2ML1 inhibits diverse proteases and particularly, we have shown that α2ML1 binds to the kallikrein KLK7, a chymotrypsin-like protease important for desquamation [15]. While a role of LRP1 in the epidermis remains to be determined, it is tempting to speculate that LRP1 might have a role in the endocytosis of complexes formed by α2ML1 and proteases such as KLK7 or other members of the kallikreins.

An additional role for LRP1 in epidermis may be to regulate the activity of some lipases. It is known that LRP1 plays an important role in lipid metabolism by binding lipoproteins (or chylomycron remnants) generated by hydrolysis of triglycerides by lipoprotein lipases [6], [34], [35]. In addition, LRP1 also binds directly the lipases that process triglycerides and subsequently mediates catabolism of these lipases [36], [37], [38]. In the epidermis, extracellular lipids such as ceramides, cholesterol, fatty acids, and cholesterol esters, are essential for the barrier function (for review [39]). Of interest are three new genes we have recently identified which encode putative secreted hydrolases specifically expressed in the epidermis [40]. These newly identified lipases are strong candidates for the extracellular hydrolysis of triglycerides in the intercellular space of the stratum corneum. In the future, it will be of particular interest to analyze whether LRP1 can regulate the activity of theses lipases.

In conclusion, we have shown that LRP1 can mediate internalization of the RBD domain of α2ML1, suggesting that complexes formed by α2ML1 with proteases may undergo endocytosis via LRP1, a mechanism that could contribute to regulate desquamation. Furthermore, we revealed a new, unknown function for LRP1 in epidermis, which we assume to be crucial in view of the biological importance of this multifunctional receptor in other tissues.
